# The variety mixture strategy assessed in a G × G experiment with rice and the blast fungus *Magnaporthe oryzae*

**DOI:** 10.3389/fgene.2013.00312

**Published:** 2014-01-16

**Authors:** Romain Gallet, François Bonnot, Joëlle Milazzo, Christophe Tertois, Henri Adreit, Virginie Ravigné, Didier Tharreau, Elisabeth Fournier

**Affiliations:** ^1^Institut National pour la Recherche Agronomique, UMR BGPIMontpellier, France; ^2^CIRAD, UMR BGPIMontpellier, France

**Keywords:** G × G interactions, variety mixture, partial resistance, rice, *Magnaporthe oryzae*, rice blast disease

## Abstract

Frequent and devastating epidemics of parasites are one of the major issues encountered by modern agriculture. To manage the impact of pathogens, resistant plant varieties have been selected. However, resistances are overcome by parasites requiring the use of pesticides and causing new economical and food safety issues. A promising strategy to maintain the epidemic at a low level and hamper pathogen's adaptation to varietal resistance is the use of mixtures of varieties such that the mix will form a heterogeneous environment for the parasite. A way to find the good combination of varieties that will actually constitute a heterogeneous environment for pathogens is to look for genotype × genotype (G × G) interactions between pathogens and plant varieties. A pattern in which pathogens have a high fitness on one variety and a poor fitness on other varieties guarantees the efficiency of the mixture strategy. In the present article, we inoculated 18 different genotypes of the fungus *Magnaporthe oryzae* on three rice plant varieties showing different levels of partial resistance in order to find a variety combination compatible with the requirements of the variety mixture strategy, i.e., showing appropriate G × G interactions. We estimated the success of each plant-fungus interaction by measuring fungal fitness and three fungal life history traits: infection success, within-host growth, sporulation capacity. Our results show the existence of G × G interactions between the two varieties Ariete and CO39 on all measured traits and fungal fitness. We also observed that these varieties have different resistance mechanisms; Ariete is good at controlling infection success of the parasite but is not able to control its growth when inside the leaf, while CO39 shows the opposite pattern. We also found that Maratelli's resistance has been eroded. Finally, correlation analyses demonstrated that not all infectious traits are positively correlated.

## Introduction

In the decades following the so-called “Green Revolution,” crop production (and particularly rice and wheat) has heavily relied on the deployment of a small set of genetically related high-yield plant varieties in large monocultures, and the concomitant use of fertilizers and pesticides. The failure of some pesticides to control diseases and the need to reduce the use of chemicals have led to increased interest in the design and use of varieties exhibiting resistance to pathogens. Because of its clear resistance phenotype and dominant expression, complete resistance has long been favored by crop breeders. This type of resistance, which blocks the entry of the pathogen through a hypersensitive response (HR), is generally triggered by a specific recognition of a given pathogen genotype by the plant. This interaction is under a gene-for-gene control (Jones and Dangl, 2006). Deploying varieties with complete resistance in large-scale monocultures almost ineluctably leads to the breakdown of resistance, following adaptation of pathogen populations (McDonald and Linde, 2002). Indeed, such resistance can easily be overcome as parasites can counter-adapt by a single mutation (point mutation, deletion, or transposon insertion) in their so-called avirulence genes, making them invisible to the plant protection system (Jones and Dangl, 2006). Due to the overwhelming accumulation of examples of complete resistance breakdown (McDonald and Linde, 2002; Palloix et al., 2009), partial resistance has represented a promising alternative for a while. Partial resistance, also called quantitative resistance, is such that no HR response blocks the entry of the pathogen which can therefore infect the plant, but the expression of symptoms (damaged plant surface) stays low. Partial resistance is supposed to be non-specific and under the control of multiple QTL, (but see, Fukuoka et al., 2009) supposedly making it much harder to circumvent—therefore more durable than complete resistance (Boyd, 2006; Boyd et al., 2013). However, examples of erosion of partial resistance (Lannou, 2012) or the discovery of specific partial resistances (Young, 1996; Antonovics et al., 2011) also accumulate, challenging the hypothesis of durability of such resistance.

A promising strategy to sustainably control pathogen populations using partial resistance is to design “evolution-proof” mixtures of varieties, such that the effect of plant varieties on pathogen performance, their frequencies and their spatial arrangement would maintain the epidemic at a low level and hamper pathogen adaptation to varietal resistance. Such strategies based on spatial heterogeneity of selection pressures have already been designed and successfully applied to manage the evolution of resistance of insects to pesticides (Lenormand and Raymond, 1998) and to Bt GM-crops (Vacher et al., 2003). As repeatedly shown in theoretical models of adaptation to heterogeneous environments, the evolution of specialization can only be hampered if adaptation to one habitat trades off with adaptation to the others (Ravigné et al., 2009 and references therein). Pathogens have the intrinsic property that their habitat is a living organism. As a consequence, their fitness likely depends on both parasite and host genetic backgrounds (the so-called extended phenotype, Dawkins, 1982). Therefore, for a varietal mixture to work, a pathogen performing well in a given plant variety should have low fitness on another; in other words there should be pronounced host genotype by parasite genotype (G × G) interactions over fitness traits (Lambrechts et al., 2006).

In natural systems, the coevolutionary dynamics between host defenses and parasite weapons tends to build G × G interactions (Lambrechts et al., 2006). G × G interactions are considered as a very common feature of host-parasite relationships and were experimentally identified in several natural host-parasites systems (Lambrechts et al., 2006 and references therein). The existence of G × G interactions is thought to regulate disease prevalence and to affect the evolution of pathogens in natural host populations (Lambrechts et al., 2009; Tack et al., 2012 and references therein). In agricultural plant-parasite systems, the situation may be substantially different. First, the coevolutionary dynamics is not the same. The plant “populations” do not evolve as such, as they are sown and harvested under human control. The production and deployment of resistant varieties by crop breeders constitutes a form of evolution but it occurs at a very low rate and with many different constraints other than biological ones. Second, due to the globalization of agriculture, crop pathogens are often accidentally transported over large spatial scales (including worldwide in some crops), so that pathogen populations may be composed of strains with no evolutionary history on the considered varieties (Saleh et al., 2013). As plant varieties may also be distributed worldwide, parasites naïve to the plant variety might be encountered more often than in natural systems.

In absence of strong coevolutionary dynamics, the occurrence of G × G interactions is hard to predict. Furthermore, given the importance of pathogen dynamics of adaptation in agricultural systems (Gilligan, 2008; Pariaud et al., 2009; Lannou, 2012), untangling the effects of pathogen and host genotype on pathogen life history within the host and characterizing G × G interactions is an important step before designing “evolution-proof” landscapes using mixtures of varieties.

*Magnaporthe oryzae*, the fungus responsible for the rice blast disease, is one of the pathogens where documenting G × G interactions is most necessary. Disease control is mainly genetic, but complete resistance genes of rice varieties are frequently and rapidly overcome following the emergence of virulent blast strains (Ou, 1985), and the deployment of partially resistant varieties is already effective in regions were blast pressure is considered relatively low. The fungus originates from South-East Asia and has invaded four continents (Saleh et al., 2013). It is frequently accidentally transported over large spatial scales (i.e., worldwide) probably through exchanges of infected seeds, so that epidemics may gather genotypes from remote areas (Saleh et al., 2013).

As a first step to predicting the efficiency of rice mixtures of partial resistance to control the blast disease, it is therefore necessary to characterize how both plant variety and pathogen genotype determine pathogen performances. To do so, we inoculated 18 different *M. oryzae* genotypes, representative of the worldwide diversity of the pathogen, on three rice plant varieties showing different levels of partial resistance. Two of these varieties are moderately resistant while the third is highly resistant. We looked for G × G interactions by analyzing the fungal fitness (total number of spores on three plants from the same pot) and three traits used to estimate the success of each plant-fungus interaction: infection success (number of lesions), the within-host multiplication (lesion size), and the sporulation capacity (number of spores produced by lesion). We asked the following questions: (1) What are the infection traits under the shared control of plant and pathogens? (2) Are there G × G interactions over these traits? (3) Are these traits correlated, and correlated to fitness? (4) What can we conclude regarding the use of variety mixture in the control of the rice blast disease?

## Materials and methods

### Biological material

The worldwide genetic subdivision of *Magnaporthe oryzae* populations was shown to be strongly associated to mating type and rice variety rather than to geography (Tharreau et al., 2009; Saleh et al., 2013). Furthermore, *M. oryzae* is frequently transported over long distances through seed exchanges, and this pattern might increase in the future through intensification of globalization. We therefore assumed that any variety mixture could in principle be confronted to strains from worldwide, either now or in the future. More specifically, previous studies showed that worldwide genetic diversity of *M. oryzae* is organized in three genetic clusters (Tharreau et al., 2009; Saleh et al., 2013): one encompassing all female-fertile strains, one encompassing strains sampled on indica rice, and the last encompassing strains sampled on japonica rice. To account for such variability, we chose 18 strains of *Magnaporthe oryzae* among a core collection hosted in the laboratory, encompassing six *M. oryzae* isolates representative of each of these three genetic groups (Table S1). Strains were also chosen so that both mating types (type 1 and 2) as well as female fertile strains would be represented. Strains were stored on dry filter papers at −20°C as described in Valent et al. (1991).

The three rice varieties used in this study were (by order of increasing partial resistance): Maratelli (japonica type), CO39 (indica type), and Ariete (japonica type). The level of partial resistance was previously estimated by visual evaluation of infected leaf area in independent assays carried out in controlled conditions in our lab.

### Inoculation procedure

Asexual spores (conidia) to be used for inoculation were produced by growing the strains for 7 days at 25°C under white light (12 h/day) on rice flour agar medium (20 g rice flour, 2 g yeast extract, 15 g agar and 1 L water, with the addition of 500,000 IU of penicillin G after autoclaving for 20 min at 120°C). Rice seeds were sown in individual pots (4–5 seeds per pot). Pots were placed in a greenhouse for 4 weeks, so that plants produced 4–5 leaves. Plants were then inoculated with conidial suspensions calibrated at 20,000 conidia.ml^−1^ and supplemented with 1% gelatin, 20 ml suspension being sprayed on 9 pots of 5–6 plants (Berruyer et al., 2003). Symptoms were always examined 7 days after inoculation, as latency is highly conserved in the rice—*M. oryzae* system (Roumen and Boef, 1993).

### Experimental design and evaluation of aggressiveness traits

The experiment was a factorial design organized in nine complete randomized blocks that were set up in three successive runs of three blocks. Each block contained 54 pots (experimental units) representing all the 18 strains × 3 variety combinations, and was scored by a single evaluator. In each block, pots were randomized to avoid potential bias due to gradients of temperature, light, or moisture. Each pot contained 5–6 plants. For the evaluation of each aggressiveness traits, a single measure per pot was obtained from three plants at the same developmental stage. Symptoms were observed on one leaf of each of the three plants, either on the entire leaf (the surface being measured) or on a 5 cm central window, depending on the lesion density on the leaf. We measured the fungal fitness (total number of spores) and the three following aggressiveness traits: infection success (number of lesions), within host growth (lesion size), and the sporulation capacity (number of spores per lesion). The number of lesions and the size of each lesion were scored on each of the three leaves and the lesion size was averaged per experimental unit (9 blocks × 54 experimental units = 486 values per trait). Because measuring sporulation capacity is very time-consuming, spores were collected on only one pot per run for each of the 54 combinations. For each of the 162 experimental units retained for measuring the sporulation capacity and fungal fitness, all lesions scored on the three counted leaves were pooled together in a glass tube containing 1 ml of 0.02% Tween, and placed at 25°C during 48 h to induce sporulation. Tubes were then vortexed to detach conidia from leave fragments, and conidia concentrations were estimated using a hemocytometer.

### Statistics

Count data (number of lesions and number of spores) were analyzed with a generalized linear model and the lesion surface was analyzed with a linear model. In both cases, the general model was the following:

$$\eta_{ijk}=m+v_{i}+s_{j}+c_{ij}+b_{k}+\lambda x_{ijk}$$

where η_*ijk*_ is the linear predictor for the experimental unit receiving variety *i* and strain *j* in block *k*, *m* is a constant term, *v_i_* is the effect of variety *i*, *s_j_* is the effect of strain *j*, *c_ij_* is the interaction of variety *i* with strain *j*, *b_k_* is the effect of block *k*, *x_ijk_* is a covariate measured on the experimental unit, and λ is the covariate coefficient.

For count data, we used a Poisson quasi-likelihood model in order to take overdispersion into account. In that case, η_*ijk*_ = log (μ_*ijk*_) where μ_*ijk*_ is the expected value of the count. The covariate *x_ijk_* was the logarithm of the area from which the count was obtained, because the count resulting from a Poisson process is proportional to that area. For the number of lesions, the covariate was the logarithm of leaf surface on which lesions were counted. For the number of spores per lesion, as lesions were pooled during the sporulation step, we needed to correct the number of spores by the number of lesions sampled. Therefore, for this analysis the covariate was the logarithm of the number of lesions. For fungal fitness (total number of spores), the covariate was the logarithm of leaf surface on which lesions were counted. Finally, the lesion surface was analyzed with a linear model without covariate where η_*ijk*_ is the expected value of the analyzed variable. Heteroscedasticity was effectively compensated by a log-transformation of lesion surfaces.

Total, between-group, and within-group correlations were calculated with “fungal strain” and “plant variety” as grouping variables. This decomposition of the correlation was performed in order to verify the structure of the total correlation. Total correlations were tested using Pearson's correlation test. Between and within correlations were calculated with permutation tests. Within variety and between strain correlations are genetic correlations as they were always performed among traits on a genotype level.

All analyses were performed using the statistical software package R, version 2.14.1 (R Development Core Team, 2011). Linear and generalized models were calculated using respectively the *lm* function of the standard R package *stats* and the *glm* function of the standard R package *MASS*. Correlations were calculated using the R package *DiscriMiner* (Sanchez, 2012).

## Results

### The effect of plant variety on infection traits

**Figure 1:**
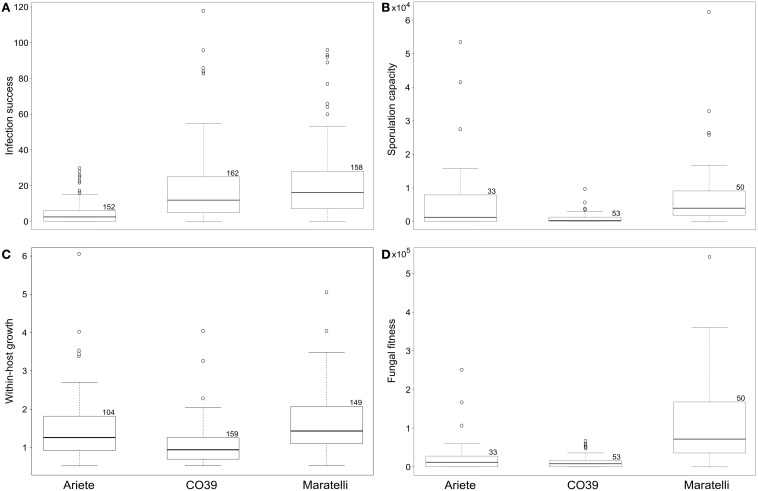
**Boxplots depicting rice varieties performance, for (A) the number of lesions, (B) the number of spores per lesion, (C) the lesion size, and (D) the number of spores per plant (our measure of fitness)**. The box limits represent the upper and lower quartiles, the bold line represents the median, and the whiskers the minimum and maximum values, excluding outliers (these latter represented by a circle). Numbers on the top right corner of boxes show the sample size. Nine independent measures per strain were used for the infection success and within-host growth, and three for sporulation capacity and fungal fitness.

Plant varieties showed significantly different levels of resistance to the fungal genotypes used in this study, on fitness and the three traits measured: infection success, within-host growth and sporulation capacity (Figure 1). No variety performed better than the others for all measured traits. As shown on Figures 1A,B,C and summarized in Table 1, Ariete was the variety best controlling the number of lesions, while CO39 was more able to control lesion size and spore production. Maratelli on the other hand, was by far the most susceptible variety for all measured traits. Overall, in terms of resistance, here defined as the ability to reduce fungal fitness, Maratelli was by far the most susceptible variety, confirming our former observations. According to the same criterion, CO39 was the most resistant. This result contradicted Ariete as being the most resistant (see Materials and Methods). This apparent contradiction comes from the fact that in previous studies, resistance was estimated by the diseased leaf area. Here, we confirm previous results since Ariete showed the most limited infected surface area, but CO39 was the best at controlling spore production. Whether diseased leaf area or sporulation control should be used as estimators of resistance is discussed later. As we were looking for variety mixtures able to control the rice blast disease on the long-term, our finding that Maratelli's resistance was circumvented by most fungal strains led us to conclude that this variety was inappropriate to be part of an “evolution proof” variety mixture. Therefore, we excluded this variety from the models looking for G × G interactions.

**Table 1:**
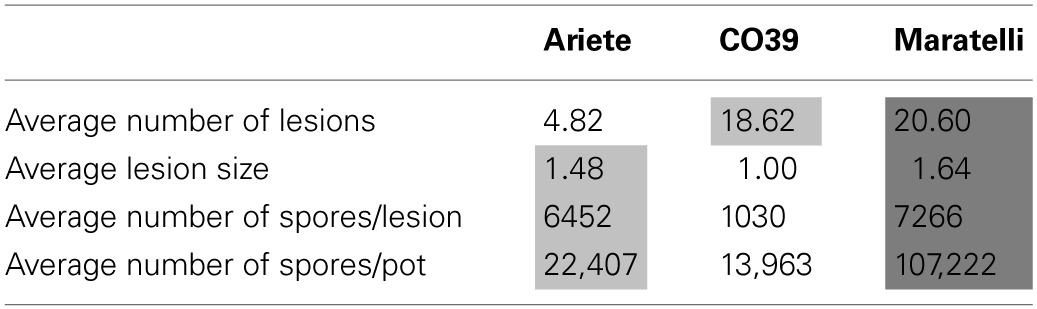
**Average performance of the three varieties on the three traits we measured**.

White, light gray, and gray cells show the most resistant, the intermediate, and the most susceptible variety on the considered trait. Data used for the calculation of these average measures are the same as those used in Figure 1.

### The effect of parasite strains on infection traits

**Figure 2:**
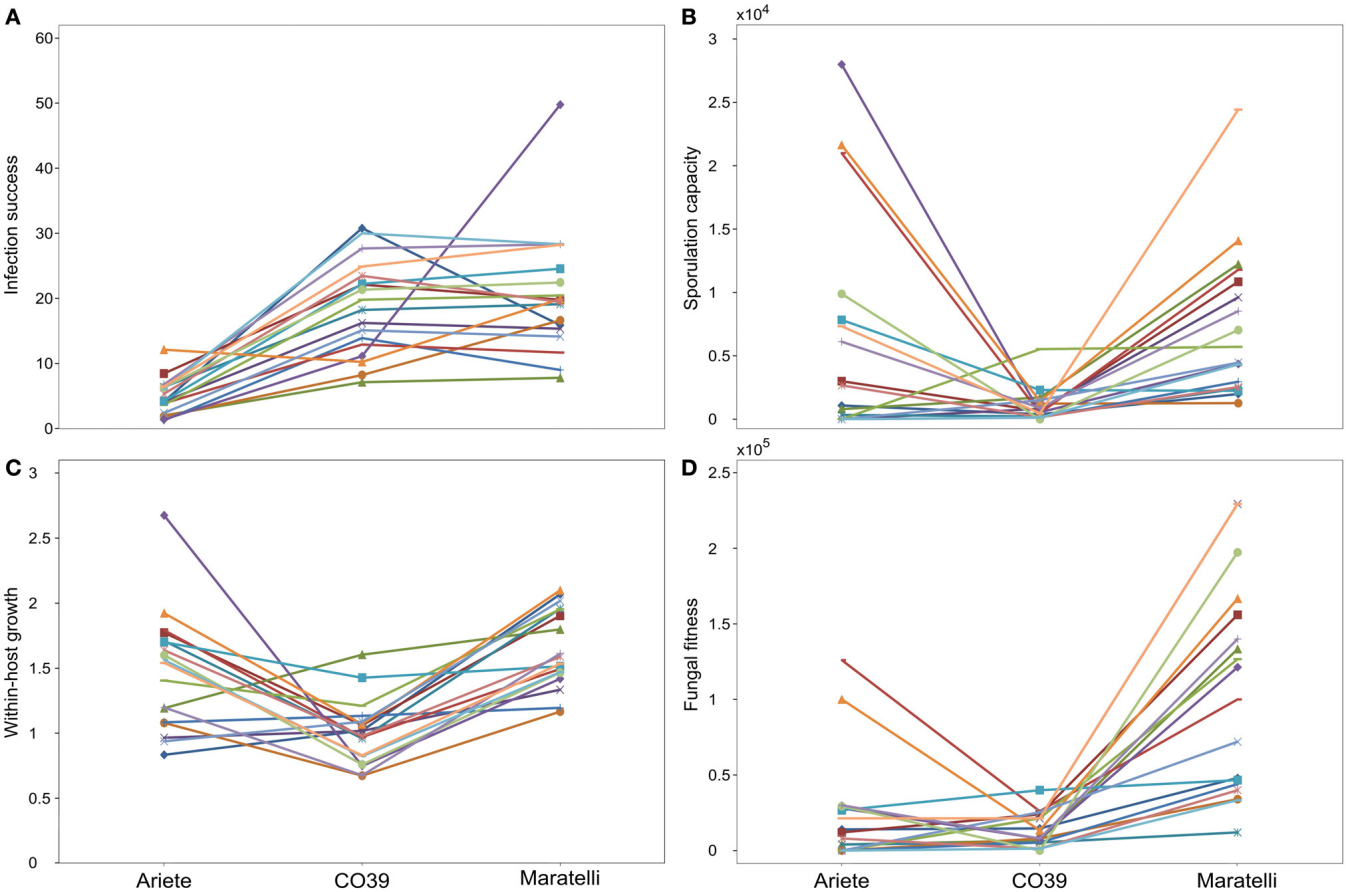
**Average performance of the 18 fungal strains on each rice varieties**. Panel **(A)** depicts infection success (average number of lesions observed on three leaves), **(B)** the sporulation capacity (average number of spores per lesion), **(C)** the within-host growth (average lesion size), and **(D)** fungal fitness (total number of spores collected on the three leaves). Points represent mean values calculated with three independent measures for sporulation capacity and fungal fitness, and nine points for the infection success and within-host growth.

In this experiment, fungal strains significantly differ in their life-history characteristics on rice plants. We observed a high variability on infection success (number of lesions) ranging from 1 to 50 lesions per plant, the within-host growth (average lesion size) ranging from 0.67 to 2.675, the sporulation capacity (the number of spores per lesion) ranging from 0 to 28,000 spores/lesion, and the fungal fitness (total number of spores produced per pot) ranging from 0 to 229,000 spores on three plants (Figure 2). We found a significant effect of the fungal genetic group [female-fertile (*F*)/sampled on Indica (*I*)/sampled on japonica (*J*)] on within-host growth (Table 2 analysis 2, with *F* > *J* > *I*), and on the sporulation capacity (Table 2 analysis 3, with *F* > *I* > *J*), as well as a marginally significant effect on fungal fitness (Table 2 analysis 4, with *F* > *I* > *J*). In all our analyses, no effect of the mating type was detected on any of these traits (data not shown).

**Table 2:** **Statistical analyses**.

**1. ANALYSIS OF THE INFECTION SUCCESS (NUMBER OF LESIONS) WITH A GENERALIZED LINEAR MODEL ON ARIETE AND CO39**						
	***Df***	**Deviance**	**Resid. *Df***	**Resid. Dev**	***Pr*(>Chi)**	
NULL			313	5066.4		
Block	8	1540.65	305	3525.8	<2.2e-16	^***^
Log(leaf surface)	1	441.37	304	3084.4	<2.2e-16	^***^
Fungi	17					
Group	2	15.87	302	3068.5	0.23506	
Strain	15	456.19	287	2612.3	1.77E-11	^***^
Variety	1	992.88	286	1619.4	<2.2e-16	^***^
Fungi × variety	17	175.22	269	1444.2	0.01516	^*^
**2. ANALYSIS OF THE WITHIN-HOST GROWTH (LESION SIZE) WITH A LINEAR MODEL ON ARIETE AND CO39**						
	***Df***	**Sum Sq**	**Mean Sq**	***F*-value**	***Pr*(>*F*)**	
Block	8	13.027	1.6283	10.4711	2.08E-12	^***^
Fungi	17					
Group	2	1.694	0.8468	5.4454	0.004921	^**^
Strain	15	5.046	0.3364	2.1633	8.41E-03	^**^
Variety	1	5.518	5.5183	35.4857	1.01E-08	^***^
Fungi × variety	17	5.785	0.3403	2.1883	0.005324	^**^
Residuals	219	34.056	0.1555			
**3. ANALYSIS OF THE SPORULATION CAPACITY (NUMBER OF SPORES PER LESION) WITH A GENERALIZED LINEAR MODEL ON ARIETE AND CO39**						
	***Df***	**Dev resid**.	***Df* resid**.	**Dev**.	***Pr*(>Chi)**	
NULL			85	835.36		
Date	2	57.787	83	777.58	0.000251	^***^
Log (number of lesions)	1	50.379	82	727.2	0.000144	^***^
Fungi	17					
Group	2	37.906	80	689.29	4.35E-03	^**^
Strain	15	298.838	65	390.45	6.15E-12	^***^
Variety	1	116.085	64	274.37	7.88E-09	^***^
Fungi × variety	16	90.08	48	184.29	0.05627	^·^
**4. ANALYSIS OF THE FUNGAL FITNESS (TOTAL NUMBER OF SPORES) WITH A GENERALIZED LINEAR MODEL ON ARIETE AND CO39**						
	***Df***	**Dev resid**.	**Df resid**.	**Dev**.	***Pr*(>Chi)**	
NULL			85	835.36		
Date	2	57.787	83	777.58	0.002345	^**^
Log (leaf surface)	1	97.659	82	679.92	6.07E-06	^***^
Fungi	17					
Group	2	28.121	80	651.8	0.052503	^·^
Strain	15	261.66	65	390.14	1.90E-06	^***^
Variety	1	0.259	64	389.88	8.16E-01	
Fungi × variety	16	129.343	48	260.53	0.040296	^*^

The factor “Fungi” was decomposed in strain genetic group (Group) and fungal strains within group (Strain). Analyses 1 and 2 performed on Ariete and CO39 only (excluding Maratelli), are shown in Supplementary Materials. The results of statistical tests performed on these correlation coefficients are symbolized with stars (

*** p-value between 0 and 0.001

** p-value between 0.001 and 0.01

* p-value between 0.01 and 0.05

· p-value between 0.05 and 0.1).

### Correlation between traits

We looked at total and genetic correlations between the three traits: infection success, within-host growth, and sporulation capacity. We found a positive correlation between within-host growth and sporulation capacity (Table 3—*r* = 0.59, *p* = 2.15 10^−14^), but no significant correlation between infection success and sporulation capacity (*r* = −0.09, *p* = 0.295) or the growth within the host (*r* = 0.0077, *p* = 0.93).

**Table 3:** **Correlation table on the three varieties Ariete, CO39, and Maratelli**.

	**Fungal fitness**			**Sporulation capacity**			**Within-host growth**		
Infection success	Total	0.34	^***^	Total	−0.09	NS	Total	0.01	NS
	Within variety	0.25	^**^	Within variety	−0.1	NS	Within variety	0	NS
	Between fungal strain	−0.38	NS	Between fungal strain	−0.56	^*^	Between fungal strain	−0.52	^*^
Within-host growth	Total	0.53	^***^	Total	0.60	^***^			
	Within variety	0.31	^**^	Within variety	0.44	^***^			
	Between fungal strain	0.46	^·^	Between fungal strain	0.69	^**^			
Sporulation capacity	Total	0.73	^***^						
	Within variety	0.71	^***^						
	Between fungal strain	0.90	^***^						

Data used (log-transformed values) corresponded to the experimental units (pots) retained for measuring the sporulation capacity and fungal fitness and that had no missing data (136 points). Total, within-variety and between-strain correlations were calculated separately with “fungal strain” and “plant variety” as grouping variables. The number of points in the three “plant variety” groups were 33 (Ariete), 53 (C039), and 50 (Maratelli). The number of points in the 18 “fungal strain” groups was comprised between 5 and 9. Between-varieties correlations should be considered with caution because they were estimated with only three group centers. Significance level:

*** p-value between 0 and 0.001

** p-value between 0.001 and 0.01

* p-value between 0.01 and 0.05

· p-value between 0.05 and 0.1

NS, non-significant.

**Figure 3:**
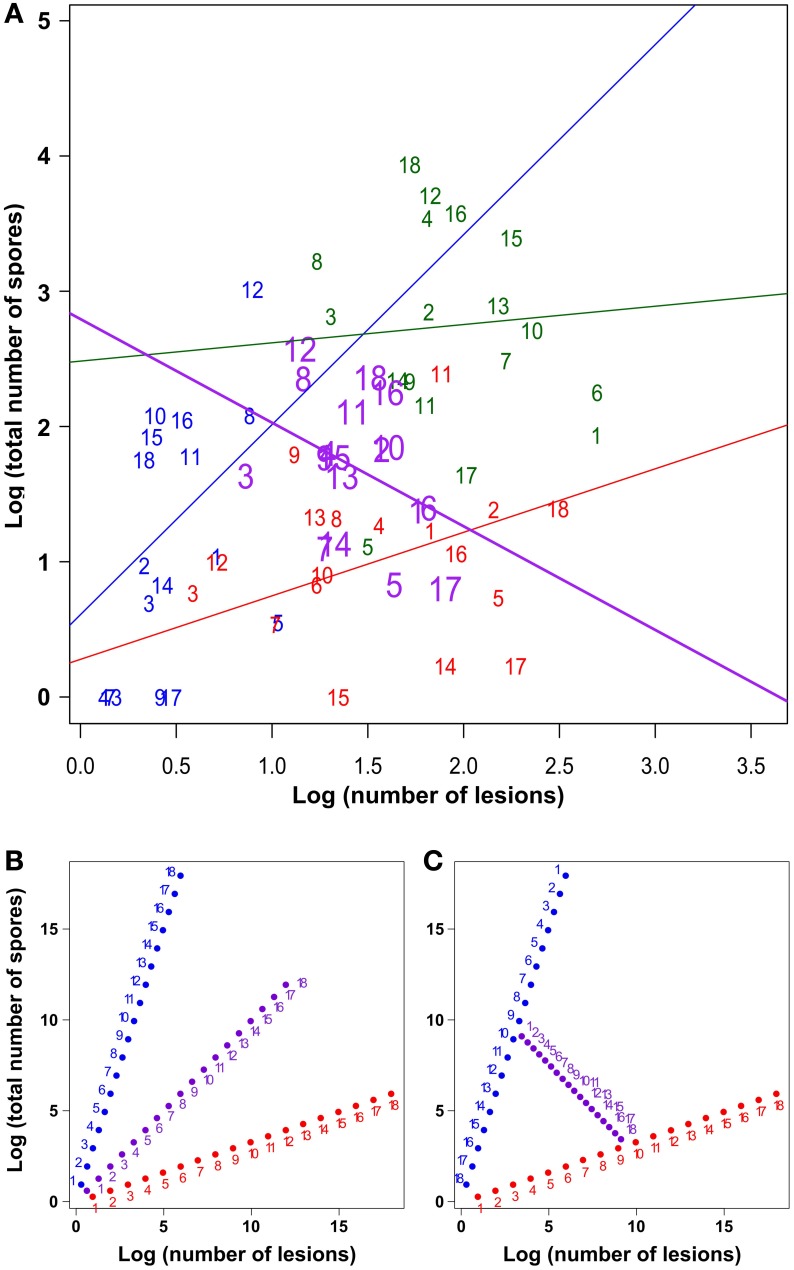
**Illustration of the within-variety and inter-fungal strain correlations between infection success (number of lesions) and fungal fitness (total number of spores)**. The panel **(A)** shows averaged measures (three replicate measures per strain per variety) of the number of lesions and the total number of spores. Numbers designate fungal strains, blue, red, and green point represent data collected on Ariete, CO39, and Maratelli, respectively. Big purple symbols represent the averaged performance of the 18 fungal strains, on the three varieties, and correspond to the data used to calculate the between-fungal strain correlation. Lines represent linear regressions. Panels **(B,C)** illustrate the different between-fungal strain correlations (purple points) that are expected **(B)** when fungal performances on two varieties are positively correlated or **(C)** when there are G × G interactions i.e., strains performing well on variety 1 perform badly on variety 2. Numbers identify fungal strains, and colors the two varieties (blue = variety 1, red = variety 2). Purple points are the mean values of both the values obtained for variety 1 and 2, data used to calculate inter-fungal strain correlation (purple points are equivalent to big purple symbols on panel **A**).

Surprisingly all total correlations implicating infection success turned out to be negative when calculated between strains (infection success vs. fungal fitness: *r* = −0.38, *p* = 0.13, infection success vs. sporulation capacity: *r* = −0.56, *p* = 0.02, and infection success vs. within-host growth: *r* = −0.52, *p* = 0.03). When the variety Maratelli was removed from the dataset (Table S3) the correlations between infection success and sporulation capacity and between infection success and within-host growth turned to be negative as well (*r* = −0.24, *p* = 0.027, *r* = −0.37, *p* = 4.65 10^−4^, respectively). The Figure 3 illustrates one example of between-strains and within-variety correlations.

Then, we looked at correlations between these three traits and fitness. Fitness was estimated by the total number of spores per pot (total number of lesions on three plants × number of spores per lesion), so we expected fitness to be positively correlated to these two traits. However, there are many ways to produce a lot of spores, for instance by making a lot of low productive lesions, or by making a few very productive lesions. Therefore, we had no *a priori* on the strength of these correlations. We observed that fitness was positively correlated to all traits, but the sporulation capacity was the trait the most correlated to fitness (fungal fitness vs. infection success: *r* = 0.34 *p* = 4.32 10^−5^, fungal fitness vs. within-host growth: *r* = 0.53 *p* = 4.12 10^−11^, fungal fitness vs. sporulation capacity: *r* = 0.73 *p* < 10^−14^). Interestingly, the correlation between fungal fitness and within-host growth or infection success dropped to 0.31 (*p* = 0.002) and 0.25 (*p* = 0.003) respectively in the within-variety analysis, contrary to the correlation between fitness and sporulation capacity, which stayed constant (*r* = 0.71, *p* = 0.001).

### G × G interactions

As pointed above, we looked for G × G interactions with Ariete and CO39 only, Maratelli being excluded from these analyses since its resistance was obviously overcome. Significant interaction between fungal strains and plant varieties were found for the infection success (Figure 2A, Table 2 analysis 1—fungi × variety *p* = 0.015) the within-host growth (Figure 2C, Table 2 analysis 2—fungi × variety *p* = 0.005) and fungal fitness (Table 2 analysis 4—Fungi × variety *p* = 0.04). On the sporulation capacity the G × G interaction was only marginally significant (Table 2 analysis 3—Fungi × variety *p* = 0.056). These interactions demonstrate that these traits are not only controlled by the parasite and the host, but by specific combinations of host and parasite genotypes.

## Discussion

In the present article, we looked at the existence of G × G interactions between rice varieties and *M. oryzae* as a first step to create rice cultivar mixtures able to control the rice blast disease. We measured key traits of the infection process and fitness of a worldwide collection of fungal strains on three popular and partially resistant rice varieties. The three traits were infection success (number of lesions), within-host growth (lesion size) and sporulation capacity (the number of spores produced per lesion).

### Varieties with contrasted resistance levels

All three traits were significantly dependent on plant varieties, confirming that these three varieties have different levels of resistance to fungal infection (Figure 1). Interestingly, no variety performed better than the others for all measured traits. CO39 was better at controlling the fungal within-host growth and the sporulation capacity than Ariete and Maratelli, while Ariete was better at limiting infection success (Table 1). However, Maratelli was by far the less resistant on all traits. Two main conclusions can be drawn from these results. Firstly, Maratelli's partial resistance has been eroded. This variety was susceptible to most of the 18 strains tested. Secondly, Ariete and CO39 have different resistance mechanisms. Ariete greatly prevents *M. oryzae* from making lesions, but appears to be unable to control its growth after successful penetration of the fungus in the leaf. This strategy results in few large lesions. CO39 on the other hand poorly controls the ability of *M. oryzae* to infect leaves, but greatly precludes fungal growth inside plant tissues. This strategy results in many small lesions. Overall, these two resistance strategies have a similar effect on fungal fitness (average fungal fitness on CO39: 1.4 10^4^ spores/plant, on Ariete: 2.2 10^4^ spores/plant = 1.6 fold higher than on CO39), meaning that both plants can roughly control fungal epidemics to the same extent. Interestingly, Ariete is considered by phytopathologists as the most resistant out of the three varieties we tested here (based on the criterion of infected leaf area), while here we consider CO39 as the most resistant variety based on its ability to limit *M. oryzae* sporulation. Obviously there are different definitions of resistance based on the scientific question asked. For phytopathologists who are interested by the damage imposed by the pathogen to the plant, measuring the infected leaf surface (success of infection × within-host growth) appears to be an appropriate measure, even though the correlation between infected leaf area and plant fitness may not be straight forward. For ecologists and epidemiologist wondering if a pathogen will create an epidemic, we believe that resistance should be defined as the ability to limit pathogen reproduction, especially in a multi-cycle disease as is rice blast. In that case, fungal fitness should be measured to estimate resistance.

### Extensive variability for life history traits among parasite strains

We found that all aggressiveness traits differed significantly among fungal strains that uncover an important genetic variability among strains of *M. oryzae* for the three life-history characteristics measured. The high phenotypic variability observed between fungal strains implies that populations composed of several strains have a high adaptive potential, with a high probability that at least one fungal strain will cause the rice blast disease. Such variable fungal populations could be a real issue when rice is grown in monoculture. Whether mixtures of varieties can control such highly adaptive populations depends on the existence of G × G interactions, a point discussed further in following sections.

We also detected significant effects of the fungal genetic group. This result shows that varieties can sometimes be preferentially susceptible to a specific fungal genetic group, which could indicate a pattern of specialization of fungal strains from a particular genetic group on specific cultivars. However, since we only studied three varieties we cannot conclude on the generality of this result.

### Correlation between traits

In this experiment, we measured three traits at different steps of the infection process, but are these traits independent or all correlated and just different ways to measure a more global trait? We found a positive correlation between within-host growth and sporulation capacity (Table 3). This shows that the bigger the lesion, the more spores are produced, an expected result already observed in previous studies (reviewed in Lannou, 2012). It would be interesting to study this relationship in more details, and determine if the number of spores produced only depends on the lesion surface or if the number of spores produced per mm^2^ can also vary. Unfortunately, in this dataset we only have average measures of lesion size and number of spores per lesion, estimating the number of spores per mm^2^ would therefore potentially be subjected to multiple biases (confounding variables). The only way to properly estimate spore density per surface unit, would be to measure the surface and the number of spores produced by single lesions.

We did not find correlations between these two traits (within-host growth and the sporulation capacity) and infection success (Table 3). However, these correlations turned out to be negative when calculated between strains (Figure 3). Such negative correlation means that on average (on the three varieties), fungal strains making a lot of lesions produce few spores and *vice versa*. Rather than evoking a trade-off in *M. oryzae* between infection success and other traits, we believe these negative correlations constitute another demonstration of the existence of G × G interactions. In other words, if there is a trade-off in *M. oryzae*, it is probably not between infective traits, but between performances on the two host varieties. As shown on Figures 3B,C, if the performance of fungal strains on different varieties is positively correlated, then the between-fungal strain correlation, should be positive as well (Figure 3B). However, if there are G × G interactions, i.e., if strains performing well on the first variety perform badly on the other variety, the slope of the between-fungal strain correlation should be negative (Figure 3C). Thus, our finding that the correlations between within-host growth or the sporulation capacity and infection success are neutral at the within variety level, but negative at the inter-fungal strain level, shows that: (1) these traits are not correlated, and (2) support our results on the existence of G × G interactions, obtained in independent statistical models.

### Estimating fitness in the rice—*M. oryzae* system

To our knowledge, this study is the first attempting to measure fungal fitness (here defined as the total number of spores produced by a given genotype) in the rice-*M. oryzae* system. Some data are available on lesion number and lesion size but seldom on spore production *in planta* (Talukder et al., 2004). As we discussed in the previous paragraph, not all traits measured are positively correlated. Therefore, we can ask the questions (1) do all these traits correlate positively to fungal fitness, (2) is there a trait that we can use as a proxy of fitness?

We observed a positive correlation between fitness and all three traits, although these correlations varied in strength. We found out that infection success and within-host growth were poorly correlated to fitness (Table 3) as compared to sporulation capacity, which was highly correlated to fitness. This correlation analysis thus indicates that sporulation capacity is the most appropriate trait to estimate *M. oryzae* fitness when growing on rice. But, whatever its relevance as a fitness proxy, this trait may not be the easiest way to estimate fitness. In the rice—*M. oryzae* system, measuring the number of spores per lesion is far from simple, very time consuming, and most importantly as complicate as direct fitness measurement, which requires sampling the entire spore population from the plant population. Therefore, we believe that the best way to measure fitness may simply be to collect all of the spores, rather than estimating it with proxies.

### G × G interactions

Predicting the long-term response of *M. oryzae* strains to a mixture of rice varieties requires studying G × G interactions over epidemiological traits involved in the association (Restif and Koella, 2004; Lambrechts et al., 2006). Different kinds of G × G interactions exist, but only a specific one guarantees an efficient disease control on the long-term, the one in which the pathogen's performance on one host genotype trades offs with its performance on other host genotypes (Figure 1c in Lambrechts et al., 2006).

To our knowledge, only few studies have investigated G × G interactions in plant-pathogen systems with quantitative epidemiological traits (Van Ginkel and Scharen, 1988; Kaltz and Shykoff, 2002; Salvaudon et al., 2007; Bruns et al., 2012; Pariaud et al., 2012). Interestingly, in these five studies (all performed on different biological systems), only two observed substantial and significant G × G interactions. Salvaudon et al. (2007) observed G × G interactions between *Hyaloperonospora arabidopsis* and *Arabidopsis thaliana* on both traits they measured. Pariaud et al. (2012) observed G × G interactions between latent period and spore production capacity in the wheat—*Puccinia triticina* system. Besides the fact that the existence of G × G interactions may obviously depend on the pathosystem considered, this also underlines that such interactions are very difficult to detect. This could be due to a lack of statistical power, to masking effects such as the presence of very susceptible hosts in the dataset, high migration rates or high levels of host local diversity (precluding parasite's specialization). With the rice—*M. oryzae* system, few studies have performed experiments allowing to test for G × G interactions for partial resistance, i.e., challenging various fungal strains on panels of rice varieties (Bonman and Bandong, 1989; Roumen, 1992). Unfortunately, these studies only looked at one qualitative or quantitative trait and G × G interactions were only formally addressed in Roumen (1992) which showed a small interaction on the number of sporulating lesions.

In our statistical analyses on Ariete and CO39, significant G × G interactions where detected on all variables. Our correlation analyses (see previous section) showed that the inter-strain correlation between epidemiological traits were negative (e.g., Figure 3). These results confirm our visual examination (Figure 2) that the average performances of fungal strains on one variety, trade-off with the average performances observed on the other variety. This pattern corresponds to the kind of G × G interactions required to control the evolution of the parasite.

In our experiments, strains and varieties were sampled independently. Therefore, they did not co-evolve with each other, but rather represent naïve interactions. In this context, the detection of G × G interactions indicates that fungal strains were probably already adapted to overcome a specific resistance. As we found out that Ariete (japonica) and CO39 (indica) have two different resistance strategies, we asked the question whether strains sampled on japonica varieties were more prone to overcome Ariete's resistance, and strains sampled on indica varieties prone to overcome CO39's resistance. To test this hypothesis, we built statistical models in which the “fungal strain” variable was replaced by the “strain genetic group” variable. We failed to detect an effect of this factor or G × G interactions in these analyses (except for within-host growth, Table S2 analysis 2). These results do not support our hypothesis that fungal strains sampled on a particular variety were more prone to overcome a specific plant resistance. Preformed defense was shown to be a major component of partial resistance to blast of tropical japonica rice varieties (Vergne et al., 2010) but not in other genetic groups. But, it has to be noted that we have no information on the diversity of resistance strategies in the rice japonica and indica subspecies, and it is possible that these different resistance strategies can be found in both indica and japonica. Clearly, understanding the nature of these G × G interactions certainly requires extended experiments performed on more rice varieties belonging to the indica and japonica subspecies.

### Variety mixture as a strategy to limit parasite evolution

The mixture of varieties is a seductive strategy to control crop diseases. It has been used at many occasions, especially on cereal crops (see Mundt, 2002, for a review). Surprisingly such strategy has only been tested a few times to control asexual pathogens and more specifically the rice blast disease (Zhu et al., 2000; Castilla et al., 2010; Raboin et al., 2012).

Key factors involved in variety mixture are the spatial distribution of varieties, the epidemiology of the pathogen, and of course, the mixture composition. Like in most variety mixtures, the trials performed on the rice blast disease used resistant and susceptible varieties. In such settings, no G × G interactions are involved, the resistant variety protects the susceptible one, but this protection will fail the day a mutant pathogen overcoming the resistance will appear (by spontaneous mutation or by migration). Thus, because of their composition, most variety mixtures are designed to delay the erosion of resistance. However, this “slowing evolution down” effect might be thwarted by the world globalization favoring pathogen migration. The selection of pathogens able to overcome complete resistances might appear at a fast pace, even when variety mixtures are used.

The ideal situation to control crop diseases on the long-term would be to design variety mixtures such that the pathogen cannot adapt to all varieties, because of the existence of trade-offs. Pathogens, like any other living organisms, are subjected to trade-offs limiting their host spectrum to a specific set of host genotypes. The existence of these trade-offs implies that when a pathogen adapts to a new host, it loses its ability to infect another host. Designing such “evolution proof” mixtures robust to parasite evolution can hardly be achieved with complete resistance varieties. Indeed, these resistances are easily eroded and there is not much evidence supporting the existence of cost of virulence associated to these resistances (Parlevliet, 1981; Huang et al., 2006).

However, the use of partially resistant varieties appears to be promising in finding such an “evolution proof” mixture. Contrary to complete resistance, partial resistances generally involve many genes, which greatly increase the chances to involve trade-offs. Furthermore, partial resistances are effective on different pathogen species and genotypes within these species (Poland et al., 2009), contrary to complete resistance which are very specific. Evolutionary proof mixtures are characterized by G × G interactions between plants and pathogens, due to the fact that when a strain is adapted to a particular variety, it suffers a poor fitness on the other.

In this study, we looked for G × G interactions between various *M. oryzae* strains and three partially resistant varieties, in order to identify candidate varieties that could be used in “evolution proof” mixtures. Four potential mixtures can be made out of these three varieties: the three combinations of two varieties, and the mixture of all varieties together. We found out that the resistance of Maratelli was eroded, and for this reason we considered all mixtures including this variety as inappropriate for the control of the rice blast disease. The presence of a susceptible variety in a mixture would greatly increase the population size of the parasite, causing more disease and increasing the probability to select more aggressive parasites than in a monoculture of a partially resistant variety. The only mixture left to test was the Ariete—CO39 mixture. We detected significant G × G interactions indicative of a trade-off between varieties for the parasite on all infectious traits and fungal fitness when considering this mixture (see above). Thus, it seems that the Ariete—CO39 mixture, would introduce some variability in the biotic environment of the fungus, representing a challenge during the adaptation of the parasite. From these results, we believe that, these two varieties, or two varieties having different partial resistance strategies, could be used in a variety mixture setting.

Two kinds of outcomes are expected, when a variety mixture is used to control a plant disease. The first is the maintenance of the “specialization” pattern initially observed with the G × G interactions; parasite populations evolve to specifically exploit a particular variety of the mixture. In that case, since only some of the plants from the mixture are suitable hosts, the disease level should remain low. The second expected outcome is the evolution of a generalist genotype that would perform well on both varieties. In this context, the use of variety mixture would be useless. Our finding of a trade-off between *M. oryzae*'s performance on the two different varieties is a promising result indicating that the parasite would have a hard time to adapt to both Ariete and CO39. Whether *M. oryzae* will only adapt on specific varieties or become generalists requires more experiments. Performing serial passage experiments of *M. oryzae* on single varieties or mixture of varieties should provide insightful results regarding the ability of this parasite to adapt or not to different varieties, and regarding the efficiency of variety mixtures.

## Conclusion

In this work we looked for G × G interactions between rice varieties and *M. oryzae* in order to identify combinations of varieties that could be used in a variety mixture strategy aiming at controlling the rice blast disease. We did observe G × G interactions on all the traits we measured when considering Ariete and CO39, indicating that a mixture between these two varieties would be *a priori* successful. These G × G interactions were driven by the existence of two different resistance strategies in these varieties, creating an unstable adaptive landscape for the parasites. Thus, in theory, variety mixture in which different partial resistance strategies are used could be used to control the rice blast disease. Finding the right variety combination requires identifying varieties with different partial resistance, similar phenology, and agronomic properties so the seeds can actually be mixed, grown, and harvested together. Whether generalist parasite genotypes able to overcome both strategies will evolve remains to be tested in serial passage experiments, before thinking of applying this variety mixture strategy in the field.

### Conflict of interest statement

The authors declare that the research was conducted in the absence of any commercial or financial relationships that could be construed as a potential conflict of interest.

## Supplementary material

Table S1 **List of the fungal strain used in this study**.

Table S2 **Statistical analyses**.

Table S3 **Correlation table**.
