# Supplementary material for: The variety mixture strategy assessed in a G × G experiment with rice and the blast fungus Magnaporthe oryzae
Source: Front Genet. 2014 Jan 16;4:312. doi: 10.3389/fgene.2013.00312 (PMC3893683; doi:10.3389/fgene.2013.00312)
Supplement: Table S1 — List of the fungal strain used in this study. [file Presentation1.PDF]

Supplementary materials

Table S1 - List of the fungal strain used in this study

Strain number	Strain name	Mat type	Group[1]	Isolated on variety	Country
1	BR0019	2	I	Tetep (indica)	Brasil
2	BR0026	1	J	Irat177 (tropical japonica)	Brasil
3	CD0101	2	I	BG90-2 (indica)	Ivory Cost
4	CH0052	1	J	B40 (indica)	China
5	CH0063	1	J	nd	China
6	CH0533	2	F	nd	China
7	CM0028	2	I	ITA212 (Indica)	Cameroon
8	FR0127	1	J	Faraman (temperate Japonica)	France
9	IN0072	1	J	HR12 (indica)	India
10	JP0010	1	F	Sasanishuki (temperate Japonica)	Japan
11	MD0116	2	I	nd	Madagascar
12	MD0929	2	I	Fofifa161 (tropical japonica)	Madagascar
13	PH0019	2	I	Milyang49 (indica)	Philippines
14	PR0009	1	J	Ringo (temperate Japonica)	Portugal
15	TH0012	1	F	Barley	Thailand
16	TH0016	2	F	Barley	Thailand
17	US0041	2	F	nd (temperate Japonica)	USA
18	VT0003	2	F	Nep Lun (temperate Japonica)	Vietnam

Table S1: List of the 18 fungal strains used in this study.

[1]: Tharreau et al. (2009)

Table S2 - Statistical Analyses

Here are presented statistical models performed on Ariete and CO39 with the factor "strain genetic group" (Group) instead of "fungal strains" (Strain).

1. Analysis of the number of lesions with a generalized linear model

	Df	Deviance	Resid. Df	Resid. Dev	Pr(>Chi)	
NULL	313	5066.4				
Block	8	1540.65	305	3525.8	< 2.2e-16	***
Log(leaf surface)	1	441.37	304	3.08E+03	4.02E-14	***
Variety	1	940.79	303	2143.6	< 2.2e-16	***
Group	2	27.91	301	2.12E+03	0.1641	
Fungi x variety	2	4.02	299	2111.6	0.7706	

2. Analysis of the lesion size with a linear model

	Df	Sum Sq.	Mean Sq.	F value	Pr(>F)	
Block	8	13.027	1.6283	9.8725	6.46E-12	***
Variety	1	5.837	5.8369	35.389	9.10E-09	***
Group	2	1.982	0.991	6.0086	0.002828	**
Fungi x variety	2	3.211	1.6056	9.735	8.50E-05	***
Residuals	249	41.069	0.1649			

3. Analysis of the number of spores per lesion with a generalized linear model

	Df	Dev resid.	Df resid.	Dev.	Pr(>Chi)	
NULL	85	835.36				
Date	2	57.787	83	777.58	0.010852	*
Log (number of lesions)	1	50.379	82	727.2	0.004979	**
Variety	1	203.814	81	523.38	1.62E-08	***
Group	2	28.268	79	495.12	0.109396	
Fungi x variety	2	27.093	77	468.02	0.119942	

4. Analysis of the number of spores per plant with a generalized linear model

	Df	Dev resid.	Df resid.	Dev.	Pr(>Chi)	
NULL	85	835.36				
Date	2	57.787	83	777.58	0.0744	.
Log(leaf surface)	1	35.126	82	742.45	0.07552	.
Variety	1	33.808	81	708.64	0.08123	.
Group	2	25.249	79	683.39	0.32134	
Fungi x variety	2	5.992	77	677.4	0.76381	

Table S3 - Correlation table

	Fungal fitness			Sporulation capacity			Within-host growth		
Infection success	Total	0.19	.	Total	-0.24	*	Total	-0.37	***
	Within variety	0.36	***	Within variety	0.03	NS	Within variety	-0.02	NS
	Between fungal strain	-0.13	NS	Between fungal strain	-0.34	NS	Between fungal strain	-0.25	NS
Within-host growth	Total	0.39	***	Total	0.66	***			
	Within variety	0.39	***	Within variety	0.56	***			
	Between fungal strain	0.49	*	Between fungal strain	0.67	**			
Sporulation capacity	Total	0.70	***						
	Within variety	0.71	***						
	Between fungal strain	0.85	***						

Table S3: Correlation table on the varieties Ariete and CO39 (excluding Maratelli). Data used (log-transformed values) corresponded to the experimental units (pots) retained for measuring the sporulation capacity and fungal fitness and that had no missing data (86 points). Total, within-variety and between-strain correlations were calculated separately with "fungal strain" and "plant variety" as grouping variables. The number of points in the two "plant variety" groups were 33 (Ariete) and 53 (C039). The number of points in the 18 "fungal strain" groups was comprised between 5 and 9. Note that correlations within and between varieties could not be calculated as in this dataset, only two varieties are considered. Significance level: *** = 0.001, * = 0.05, • = 0.1, NS= non significant.

Bibliography

Tharreau, D., Fudal, I., Andriantsimialona, D., Utami, D., Fournier, E. & Lebrun, M. (2009). World population structure and migration of the rice blast fungus, *Magnaporthe oryzae*. In: *Adv. Genet. genomics Control rice blast Dis.* (eds. Wang, G.-L. & Valent, B.). Springer Netherlands, Dordrecht, pp. 209–215.
